# Vaccine-Associated Enhanced Respiratory Disease: From Animal Models to Human Health

**DOI:** 10.3390/vaccines14090799

**Published:** 2026-09-10

**Authors:** Jacob A. Dillard, Reina A. Saldivar, Mark T. Heise, Victoria K. Baxter

**Affiliations:** 1 Department of Microbiology & Immunology, University of North Carolina at Chapel Hill, Chapel Hill, NC 27599, USA; 2 Texas Biomedical Research Institute, San Antonio, TX 78227, USA; 3 Department of Genetics, University of North Carolina at Chapel Hill, Chapel Hill, NC 27599, USA

**Keywords:** vaccine-associated enhanced respiratory disease, respiratory syncytial virus, influenza virus, coronaviruses, inactivated vaccines

## Abstract

Vaccines are a safe and highly effective tool for protecting both humans and animals against infectious diseases. Although vaccines currently in use in humans have robust safety records, historical examples show that some vaccines have resulted in adverse outcomes, including vaccine-associated enhanced respiratory disease (VAERD). VAERD occurs when preexisting vaccine-induced immunity worsens respiratory disease, rather than conferring protection during infection. Enhanced disease in the lower respiratory tract characteristic of VAERD includes worsened clinical manifestations such as pneumonia and bronchiolitis, as well as increased pulmonary pathology in vaccinated, infected individuals compared with their unvaccinated, infected counterparts. Mechanistically, VAERD has been associated with T_H_2-mediated immunopathology or antibody-dependent enhancement and is primarily associated with specific virus families, including *Pneumoviridae*, *Orthomyxoviridae* and *Coronaviridae*. Notable cases of VAERD have been associated with a respiratory syncytial virus vaccine candidate evaluated in clinical trials in the 1960s, experimental coronavirus vaccines in animal models, and in veterinary vaccines for influenza virus in swine. Existing approved vaccines are safe and highly effective tools for protecting both humans and animals against respiratory pathogens. However, as new respiratory vaccines are developed, they must also undergo careful testing for VAERD or other adverse outcomes. This review aims to provide the field with an overview of VAERD as a resource to support vaccine development and safety studies.

## 1. Introduction

Vaccine-associated enhanced disease (VAED) is a category of immune enhancement of disease defined as “an immune response to a vaccine that is causally linked to a higher risk of adverse outcomes upon infection compared with infection without prior vaccination” [1]. In cases of VAED, preexisting antigen-specific immune memory aggravates, rather than alleviates, pathological and/or clinical outcomes during infection. Vaccine-associated enhanced respiratory disease (VAERD) is a subtype of VAED that specifically focuses on the respiratory tract. VAERD can range from subtle symptoms, such as a mild cough or low-grade fever that are only impactful at the population level, to severe clinical disease, such as pneumonia and bronchiolitis, which are more easily diagnosed and characterized in individuals [2].

Although licensed vaccines are remarkably safe, a few examples from animal models and clinical trials illustrate the consequences of vaccine-induced immunopathology in the context of vaccine failure and consequent breakthrough infection. This review explores two major examples of VAERD: that resulting from the 1960s clinical trials of the formalin-inactivated respiratory syncytial virus (FI-RSV) vaccine, and reports of VAERD in animal models following vaccination against respiratory viruses. We will additionally discuss a recent set of cases of influenza A virus (IAV)-associated VAERD in production swine. Increasing our understanding of factors that contribute to VAERD will enhance the field’s ability to continue to develop safe and efficacious vaccines against respiratory pathogens, while potentially improving public perceptions of vaccine safety.

## 2. Search Methodology

This article is a narrative review and was not conducted as a systematic review; the search described below did not follow PRISMA methodology. We assembled an initial body of literature iteratively over the course of the project by tracking citations from primary reports and review articles addressing VAED. To supplement this collection, a structured search of PubMed was performed in July 2023 using combinations of the terms vaccine-associated enhanced respiratory disease, vaccine-associated enhanced disease, enhanced respiratory disease, vaccine-enhanced disease, immune enhancement of disease, and antibody-dependent enhancement, each combined with respiratory syncytial virus, coronavirus, SARS-CoV, SARS-CoV-2, MERS-CoV, influenza, measles, dengue, and feline infectious peritonitis. We screened reference lists of retrieved articles and relevant review articles for additional sources. We updated the search in July 2026 to identify literature published after the initial search; the July 2026 update represents the date of the last search.

The authors screened titles and abstracts and reviewed full texts where relevant. We included primary studies reporting enhanced clinical disease, enhanced pulmonary immunopathology, or antibody-mediated enhancement of infection following vaccination, passive antibody administration, or prior infection in humans or in animals; mechanistic studies addressing these phenomena; and reviews, expert consensus documents, and case definitions relevant to their interpretation. Studies were considered most informative when they reported an appropriate unvaccinated or adjuvant-only comparator group alongside defined clinical, virological, and histopathological endpoints. Only publications available in English were considered. We included additional sources as needed for background on the biology of the viruses discussed, the epidemiology of the diseases they cause, or the vaccines currently in use against them.

Because we searched a single bibliographic database, did not screen in duplicate with independent reviewers, and did not conduct a formal risk-of-bias assessment, we do not claim exhaustive coverage of this literature. Statements throughout this review concerning the presence or absence of particular findings should be read as describing the studies identified by this search rather than as definitive characterizations of the published literature as a whole. We nonetheless describe our approach to make the basis for study selection transparent to readers.

## 3. Mechanisms Driving VAED and VAERD

**Figure 1:**
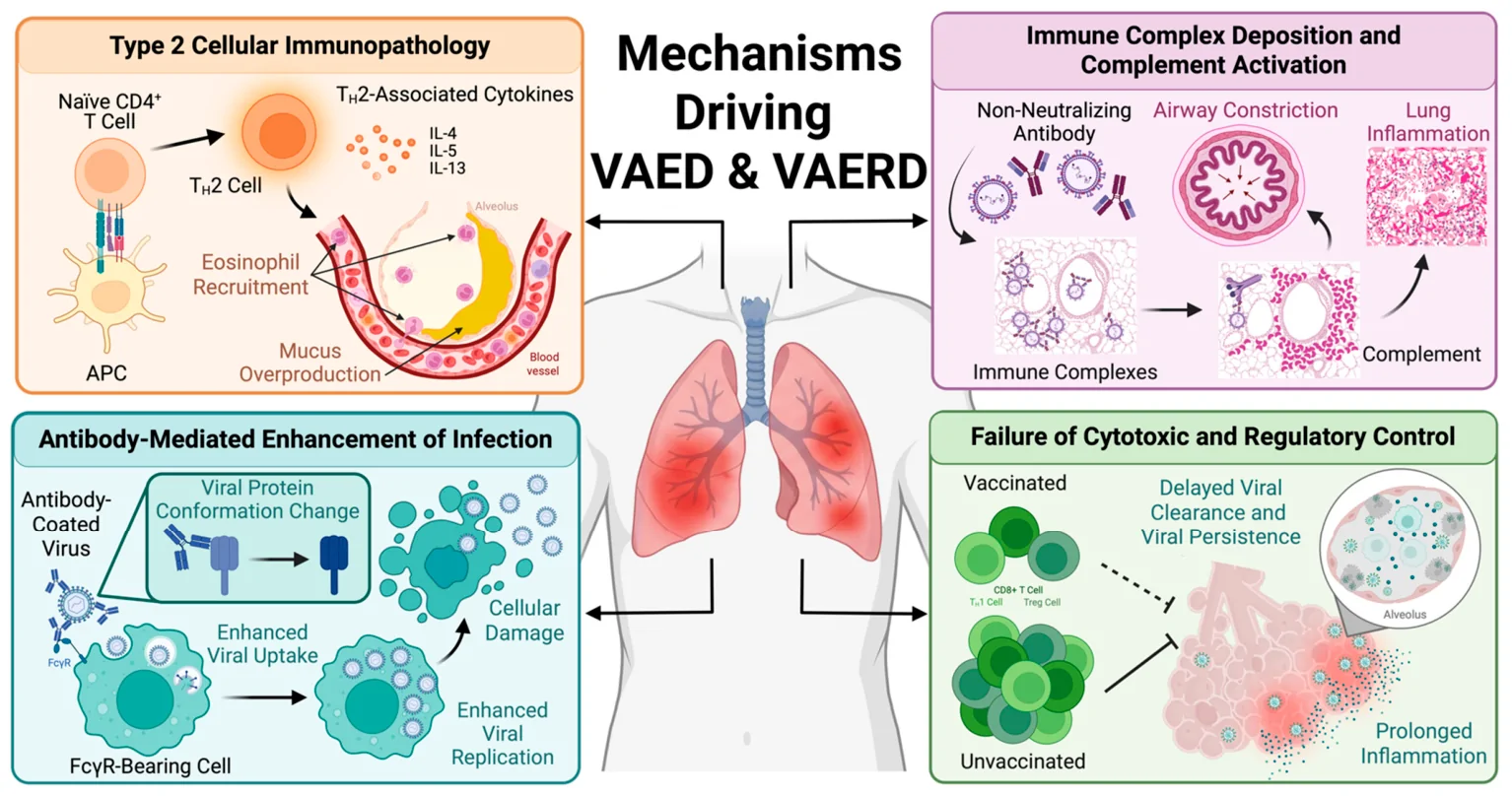
Proposed mechanisms driving vaccine-associated enhanced disease (VAED) and vaccine-associated enhanced respiratory disease (VAERD). Created with BioRender.com, accessed on 27 August 2026.

Although the VAED and VAERD examples discussed in this review arise in different host species and involve unrelated virus families, the proposed mechanisms draw on a relatively small set of immunological themes (Figure 1). Broadly, enhanced disease has been described where two conditions coincide: an immune response that fails to prevent or rapidly control infection, and a memory response that, upon antigen re-exposure, generates immunopathology in excess of that occurring in a naïve host [1,3,4,5]. Neither condition alone is generally sufficient. Vaccines that fail to protect but prime an appropriately regulated response typically result in unremarkable breakthrough infection. In contrast, vaccines that elicit durable neutralizing immunity generally prevent the antigen exposure required to drive immunopathology. It is the combination of poor protection and a pathogenic effector program that characterizes true VAED and VAERD.

### 3.1. Determinants of Poor-Quality Protective Immunity

Antigenic Conformation. Several examples of VAERD involve viral fusion glycoproteins that adopt distinct pre- and post-fusion conformations, with most potent neutralizing epitopes displayed only on the metastable pre-fusion form. Chemical or thermal inactivation can drive these proteins into the post-fusion conformation, as was demonstrated for RSV fusion (F) protein under the conditions used to produce the FI-RSV ‘Lot 100’ vaccine [6]. The resulting response may be highly immunogenic while remaining poorly neutralizing [7,8]. The original FI-RSV trials did not confirm antigenic integrity after inactivation [5,7,9,10,11], and similar considerations apply to the coronavirus spike protein and influenza hemagglutinin (HA).

Antibody Affinity and Functional Quality. Independent of conformation, the protective quality of an antibody response depends on germinal center output. FI-RSV vaccination has been shown to induce poor affinity maturation because of inadequate Toll-like receptor (TLR) stimulation, yielding antibodies with low affinity for neutralizing epitopes [12]; a comparable defect was described for formalin-inactivated measles virus (FI-MV) [13]. Consistent with this, FI-RSV formulation with a TLR9 agonist reduced immunopathologic outcomes in mice [14], and animal models with non-replicating vaccines have shown enhanced inflammation and/or disease. In contrast, replicating platforms generally provide more sustained antigen availability and innate stimulation [15].

Host Immune Status. Preexisting antigen experience appears to be protective. No child seropositive for RSV at the time of FI-RSV vaccination developed VAERD; no child developed it more than once, and FI-MV recipients who later received live-attenuated measles vaccine did not develop atypical measles [4,15,16]. Conversely, immunological immaturity may predispose infants to a naturally type 2-biased response, which may have synergized with FI-RSV priming in the original trials [17]. Maternally derived antibody has been implicated in influenza-associated VAERD in weaned piglets [18], and severe RSV disease is associated with family history of atopy and with polymorphisms in type 2 cytokine and receptor genes [5].

Vaccine Platform and Adjuvant. Whole inactivated virus-based vaccines are the most frequently represented platform among the reports surveyed here, with fewer subunit, vectored, and nucleic acid vaccines. Therefore, the antigenic complexity of inactivated vaccines, which may include non-protective antigens, may increase VAERD risk. The contribution of adjuvants has been inconsistent across studies. Alum has been associated with type 2 inflammation in RSV subunit vaccine models, an outcome avoidable through alternative adjuvant selection [5,19,20,21]. Yet, in other experimental settings, alum-adjuvanted formulations improved neutralizing antibody responses, protection, or histopathological outcomes. Adjuvants are therefore best regarded as context-dependent variables that interact with antigen, platform, host immune status, challenge strain, and dose, rather than as independent determinants of enhancement.

### 3.2. Effector Mechanisms of Immunopathology

Type 2 Cellular Immunopathology. Immunopathogenic T helper 2 (T_H_2) cells are generally regarded as central to FI-RSV VAERD and feature prominently in coronavirus vaccine–challenge models [4]. In animal models, T_H_2 responses drive eosinophil infiltration, mucus overproduction, and pneumonia; affected tissues show elevated type 2 cytokines and CD4+ infiltrates, and depletion of CD4+ cells or interleukin (IL)-4 attenuates disease [15,22,23,24,25,26,27,28,29,30]. Immunopathology is not exclusively T_H_2-mediated: distinct T_H_ subsets have been reported to mediate different components of disease, with T_H_1 responses necessary for airway obstruction and weight loss in one study [31]. Antigen modification during manufacture may promote type 2 polarization, as suggested by the observation that formalin-mediated carbonylation of RSV antigens promoted both T_H_2 skewing and VAERD in mice, an effect reduced when carbonyl groups were removed before vaccination [26].

Immune Complex Deposition and Complement Activation. When abundant non-neutralizing or low-avidity antibodies encounter high antigen loads, immune complexes may deposit in the lungs and activate complement. Post-mortem specimens from the two children who died following FI-RSV vaccination showed peribronchiolar deposition of complement component C4d, and the same study attributed airway hyperresponsiveness and bronchoconstriction to immune complex-mediated complement activation [32]; comparable findings were reported in cotton rats [4,15]. The same mechanism has been proposed in the fatal 2009 pandemic H1N1 influenza [33] and in Middle East Respiratory Syndrome coronavirus (MERS-CoV)-infected rabbits, in which passive transfer of non-neutralizing convalescent serum was sufficient to confer enhanced inflammation in naïve animals [34].

Antibody-Mediated Enhancement of Infection. In dengue virus (DENV) and feline infectious peritonitis (FIP), non-neutralizing or sub-neutralizing antibodies facilitate viral uptake into FcγR-bearing cells, increasing infection of those cells [3,35,36,37,38,39,40]. A distinct antibody-mediated mechanism operates in influenza, where antibodies binding the HA stem have been shown to increase stem flexibility and accelerate fusion kinetics, thereby promoting rather than blocking viral entry [41,42,43,44]. For coronaviruses, antibody-dependent enhancement (ADE) has been repeatedly demonstrated in vitro but has proven difficult to reproduce in animal challenge models and has not been demonstrated in humans [3,45].

Failure of Cytotoxic and Regulatory Control. Enhanced disease has also been attributed to what the response lacks. Poor induction of cytotoxic T cells, T_H_1 cells, and regulatory T (Treg) cells has been implicated in FI-RSV VAERD [4,15], and priming vaccinated animals to elicit robust cytotoxic or T_H_1 responses reduces disease during challenge [46,47,48]. VAERD in FI-RSV-vaccinated mice was associated with reduced pulmonary Treg cells, and Treg recruitment attenuated disease [49], while preexisting virus-specific cytotoxic T cells regulated T_H_ differentiation during vaccination toward a T_H_1-biased response [50]. Failure to clear virus prolongs antigen availability, sustaining the inflammatory response that produces tissue injury.

## 4. Evidence of VAED in Humans and Animals

Before surveying FI-RSV- and virus-related VAERD, it is worth briefly describing examples of VAED in animals and humans: severe dengue disease, atypical measles and feline infectious peritonitis.

### 4.1. Severe Dengue Disease

Perhaps the most famous example of immune enhancement of disease is heterologous infection with dengue virus (DENV), a mosquito-borne flavivirus that usually causes a systemic febrile illness [3,35,36,37,38,51]. Four DENV serotypes exist. Infection with one serotype confers homologous protection but is associated with an increased risk of severe disease, including dengue hemorrhagic fever and dengue shock syndrome, from infection with a different serotype. A similar pattern of disease enhancement has been observed following vaccination; clinical trials and post-marketing surveillance of Dengvaxia, a quadrivalent live-attenuated DENV vaccine, revealed an increased risk of severe dengue disease and hospitalization in vaccine recipients who were DENV-naïve at the time of vaccination [52,53,54].

However, in the context of infection or vaccination, severe DENV disease remains rare regardless of prior immune status. It appears to occur under highly specific conditions, and differentiating between immune enhancement and failure of protection is very challenging. Elucidating the relative contributions of specific mechanisms responsible for disease enhancement in human populations is extremely complex. Therefore, the role and mechanism(s) of immune enhancement during DENV infections remain unclear and are a matter of continued debate [3,37].

### 4.2. Atypical Measles

Atypical measles (AM) was a form of VAED reported in children in the 1960s [4,55,56,57,58]. Measles virus (MV) is a highly infectious airborne paramyxovirus that causes both respiratory disease and systemic illness characterized by fever, cough, sneezing, congestion, conjunctivitis, and a diffuse maculopapular rash. Measles can also present as severe disease, including pneumonia, immunosuppression, and encephalitis. Although measles is preventable by vaccination with a live-attenuated vaccine, it still causes over 100,000 deaths annually. AM occurred when children immunized with the FI-MV, which was licensed in the United States at the time, were subsequently exposed to wild-type virus during community outbreaks. Up to 60% of children immunized with FI-MV developed AM, which manifested as high fever, pneumonitis, abdominal pain, and a diffuse petechial or morbilliform rash, and often included eosinophilia (abnormally high eosinophil counts in the blood) and hepatic dysfunction [4,55,56,57,58]. Importantly, AM never recurred in children, and those initially vaccinated with FI-MV who subsequently received the protective live-attenuated measles vaccine before developing AM never developed the condition (despite abnormal local reactions to the live-attenuated vaccine itself) [4]. As we will see below, the case of AM resulting from immunization with FI-MV, as well as the likely mechanisms underlying disease enhancement, shares many features with VAERD resulting from FI-RSV [4].

### 4.3. Feline Infectious Peritonitis in Cats

Immune enhancement following vaccination is not limited to humans and is also thought to play an important role in FIP in domestic cats. Feline coronaviruses (FCoVs) usually cause asymptomatic or mild enteric disease in cats by infecting intestinal epithelial cells in the form of feline enteric coronavirus (FECV). However, in vivo acquisition of mutations in the gene encoding the viral surface glycoprotein (Spike) during infection can alter macrophage tropism and result in FIP. This systemic and usually fatal disease involves inflammation of the serous membranes lining body cavities and organs, causing peritonitis and pleuritis. Immune complexes consisting of virus and antibodies can also deposit in renal tissue, causing glomerulonephritis [39,40]. Most vaccines developed against FCoVs have failed at the preclinical stage, and some appear even to predispose recipients to enhanced disease [59].

The precise role vaccine-induced immune enhancement plays in FIP pathogenesis remains disputed, with some investigators arguing that ADE of FIPV in tissue culture and vaccine-induced FIP in cats challenged with FIPV (rather than FECV) are experimental artifacts unlikely to contribute to disease severity in natural infections. Addie, Paltrinieri, and Pedersen even suggest that laboratory demonstrations of immune enhancement have hindered FCoV vaccine development [60]. Whatever role preexisting immunity plays in driving FIP, this field demonstrates that even the perception of immune enhancement of disease and VAED can impact both our understanding of viral pathogenesis and progress toward safe and effective vaccines.

## 5. VAERD Associated with Formalin-Inactivated Respiratory Syncytial Virus (FI-RSV)

The most famous case of VAERD resulted from the FI-RSV vaccine, which underwent clinical trials in children in the late 1960s. RSV is a ubiquitous orthopneumovirus that infects ciliated respiratory epithelium and causes lower respiratory tract infections, including bronchiolitis and pneumonia involving airway hyperresponsiveness that can lead to severe bronchoconstriction and respiratory distress. The virus has a major global disease burden and is a leading cause of hospitalization in young children, immunocompromised individuals, and older adults [5,17,61,62]. RSV infection during infancy has also been implicated as a potential risk factor for asthma [19,22,62].

### 5.1. Clinical Findings

In four studies of clinical trials conducted in the United States in 1965–1967 and published in 1969, investigators evaluated the safety and efficacy of a whole virus-based FI-RSV vaccine (‘Lot 100’) formulated with aluminum hydroxide (alum) to protect children against RSV disease [7,9,10,11]. Although the vaccine was well tolerated and immunogenic, studies reported that children immunized with FI-RSV (and seronegative for RSV at the time of vaccination) tended to experience exacerbated disease during subsequent RSV community outbreaks compared with children who received control vaccines. In one report, 80% of FI-RSV vaccine recipients required hospitalization compared with 5% of control subjects. In the same study, two children in the vaccinated group died as a result of infection. However, because RSV disease can be deadly in unvaccinated groups, these deaths cannot be definitively attributed to FI-RSV [7].

### 5.2. Pathological Findings

Pulmonary pathologic examination of the two deceased subjects revealed bronchopneumonia, atelectasis, and pneumothorax [7,63]. Histopathology revealed excessive pulmonary inflammation characterized by marked polymorphonuclear infiltrates. Initial observations described ‘peribronchiolar monocytic infiltrates with some excess in eosinophils’ [7]. However, subsequent reexamination of specimens revealed predominant bronchial neutrophils, macrophages and syncytial cells, and bronchiolar neutrophils and lymphocytes, with occasional to frequent eosinophils [4,5,7,9,16,64,65]. Pure bacterial cultures were grown from pulmonary specimens obtained from the two subjects (*Escherichia coli* in one subject and *Klebsiella* in the other), suggesting possible polymicrobial infections that could complicate interpretation of cellular infiltrates and other factors underlying disease enhancement [7,19]. Signs of immune complex deposition and complement activation were later determined by detecting peribronchiolar deposition of complement component C4d, although C4d deposition can be induced by mechanisms other than complement activation [3,4,13]. Lastly, RSV was detected at high titers in the lungs of both subjects, indicating that the vaccine not only caused worse clinical disease but also failed to confer protection against the viral infection itself [4,7].

### 5.3. Epidemiology

These studies were conducted in various age cohorts ranging from 2 months to 9 years of age, and it was clear that younger age was associated with a greater risk of VAERD, with only children younger than 2 years of age who were seronegative for RSV at the time of vaccination exhibiting VAERD. Importantly, VAERD was never reported in subjects who were seropositive at the time of vaccination (and thus had presumably already been infected with RSV), and no subjects were ever reported to experience VAERD more than once. It is therefore thought that RSV infection priming protected against VAERD [4,5,62].

### 5.4. Clinical Trial Caveats and Limitations

These trials were ethically dubious for two reasons. First, they appear to have been conducted prematurely without appropriate preclinical evaluation of safety and efficacy in animal models. Although the vaccine was administered to animals in preclinical investigations, researchers did not perform challenge studies. After publication of these trial results, subsequent studies aimed at understanding the mechanisms of FI-RSV VAERD routinely reported enhanced disease in diverse animal species (to be discussed below), strongly suggesting that such observations would have been readily apparent had the appropriate studies been performed [19,66,67]. Second, the clinical trials were partially conducted in vulnerable populations; one study primarily enrolled African American children from low socioeconomic-status neighborhoods [7], and another recruited participants from a welfare institution for homeless children [11].

### 5.5. Impact on Future RSV Vaccine Development

These failed vaccine trials have profoundly shaped subsequent RSV vaccine development strategies and arguably set the RSV vaccinology field back by decades [62,63,68]. While several vaccine candidates show promising results in clinical trials and children can be passively immunized with monoclonal antibody therapy (nirsevimab and palivizumab) or through maternal vaccination during pregnancy (Abrysvo developed by Pfizer), at this time, no licensed RSV vaccine exists for children over half a century after these clinical trials were performed [64].

## 6. VAERD Associated with Vaccination Against Coronaviruses in Preclinical Animal Models

VAERD has been reported in various preclinical animal models following vaccination against several coronaviruses, as summarized in Table 1 [69,70,71,72,73,74,75,76,77,78,79,80,81,82,83,84,85,86,87]. Prior to the severe acute respiratory syndrome coronavirus 2 (SARS-CoV-2) pandemic, several preclinical studies using experimental vaccines in animal models reported that some SARS-CoV-1 and MERS-CoV vaccines caused enhanced immunopathology during viral challenge. Since the beginning of the COVID-19 pandemic, several preclinical studies have reported similar results involving SARS-CoV-2. No studies identified in the survey below reported enhanced clinical disease solely attributable to vaccines in the absence of viral challenge. Therefore, in this context, VAERD specifically refers to vaccine-associated enhanced virus-induced pathology and inflammation.

### 6.1. Clinical and Pathological Findings

Several independent research groups published these studies, which used diverse animal models and lacked standardized experimental designs or procedures. Nonetheless, numerous themes and patterns emerge from this literature. In most reports, animals showed increased pulmonary eosinophil infiltration despite neutralizing antibodies and controlled viral loads [1,88]. In addition to pulmonary eosinophilic inflammation, robust neutrophilic and lymphocytic infiltrates and enhanced type 2 cytokine production were also often described. Most studies were performed in BALB/c mice, but vaccine-enhanced immunopathology was also reported in C57BL/6 mice, hamsters, ferrets, and NHPs [1]. Type 2 inflammation was not as frequently reported in NHPs [88].

No studies summarized in Table 1 showed enhanced overt clinical disease in the same manner as the FI-RSV or FI-MV vaccines. However, it is important to recognize that animals, particularly mice, are naturally inclined to mask observable signs of illness. Dillard and Taft-Benz et al. demonstrated enhanced respiratory effort in mice following immunization with an inactivated vaccine adjuvanted with alum during heterologous challenge with a SARS-related coronavirus (SARS-r-CoV) [73]. However, researchers detected enhanced clinical disease only through whole-body plethysmography, a sensitive measure of subclinical disease that is rarely used in preclinical models. The relevance of pulmonary histopathological and inflammatory changes in the absence of enhanced clinical disease is unclear. However, relying solely on mortality and weight loss to identify VAERD may result in clinically meaningful disease being missed. In the studies summarized in Table 1, we therefore distinguish among those demonstrating enhanced clinical disease, enhanced pulmonary pathology or inflammation, and incomplete protection or breakthrough infection.

### 6.2. VAERD Associated Factors

VAERD was most commonly associated with the whole inactivated virus-based vaccine platform. However, several reports involved protein subunit vaccines and viral- or replicon-vectored vaccines, and our search identified a single report involving a nucleic acid vaccine [69,70,71,72,73,74,75,76,77,78,79,80,81,82,83,84,85,86,87]. Two independent groups showed that viral-vectored vaccines expressing the nucleocapsid (N) gene conferred poor protection and were sufficient to drive VAERD [72,86]. However, when compared to two different inactivated SARS-CoV-2 vaccines [47], the spike-encoding mRNA-1273 vaccine exhibited no collateral pulmonary pathology [74]. Furthermore, we did not identify reports of VAERD resulting from live-attenuated coronavirus vaccines. It is worth highlighting again that, in contrast to the reports summarized in Table 1, numerous other animal model studies of viral challenge involving SARS-CoV-1, MERS-CoV, and SARS-CoV-2 report protective efficacy in the absence of VAERD [1,89].

Adjuvant choice appears important; VAERD has been primarily associated with adjuvants that drive T_H_2-biased immune responses, such as alum, or with unadjuvanted vaccines, while alternative adjuvants that drive more balanced T_H_1/T_H_2 responses are often associated with improved outcomes [73,77,78]. Reports of VAERD during SARS-CoV-2 challenge included enhanced pathology in mice vaccinated with inactivated whole virus formulated with alum [74,87] and enhanced pathology in hamsters vaccinated with a suboptimal recombinant subunit spike vaccine also formulated with alum [75]. Adjuvant-dependent outcomes have also been identified in mice using a recombinant spike protein vaccine [79]. Three studies reported vaccine-enhanced immunopathology upon challenge with non-pathogenic SARS-r-CoVs [73,81,82].

### 6.3. Caveats and Limitations

Several important considerations are worth noting when considering the significance of these findings in animal models. First, no form of coronavirus disease-associated immune enhancement, whether by prior infection, vaccination, or passive administration of monoclonal antibodies or convalescent plasma, has been demonstrated in humans [1,3]. Moreover, in contrast to these few studies involving VAERD, many other studies using similar vaccine platforms and animal challenge models report protection without immunopathologic outcomes [3,88]. Because VAERD is not associated with human coronavirus vaccines and animal models do not fully recapitulate human vaccine immune responses or coronavirus-induced disease, the predictive value of animal models that report coronavirus VAERD is debatable.

Additionally, findings and interpretations in this field have been criticized for a lack of standardization in experimental design and analysis methods [2,89,90]. Wide variability in experimental models and approaches, vaccine production, and analysis and data collection makes it difficult to identify clear patterns and draw strong conclusions. Researchers have not agreed on standard positive and negative vaccine controls for comparison with experimental vaccines. However, whole inactivated virus-based vaccines formulated with alum have been used as positive controls for type 2 inflammation [2,74,88]. Although eosinophil infiltrates and type 2 cytokine production are commonly assessed, no clearly defined marker of enhancement exists, leading to inconsistent reporting of clinical, histopathological, and inflammatory outcomes. Some studies have been criticized for poor-quality histopathological data and the absence of a defined quantitative scoring system [89]. Many studies do not report whether they performed proper purification steps during vaccine production to remove tissue culture contaminants from the vaccine or the challenge virus. Because sensitization to non-viral antigens (e.g., bovine serum albumin or cellular debris) present in both vaccine and viral challenge cultures may induce inflammation, failure to purify reagents may drive pathogen-independent inflammation during challenge [2,68,89].

Lastly, because viral challenges are usually conducted shortly after vaccination, it is often unknown whether enhancement would be augmented or diminished at later time points, especially in the context of waning antibodies [2,88]. Therefore, the field would benefit from more standardization of experimental design, analysis, and reporting methods and from systematic comparisons of platforms, antigens, adjuvants, and animal species. Despite their limitations, animal models of VAERD remain useful for investigating pathophysiologic mechanisms and identifying potential indicators of suboptimal vaccine immune responses [2].

## 7. VAERD Associated with Vaccination Against Influenza Virus in Laboratory Animal Models and Commercial Swine

The World Health Organization estimates that respiratory disease caused by seasonal influenza viruses results in up to 650,000 deaths per year, primarily in individuals over 75 years of age [91]. Vaccination against influenza virus in humans is primarily accomplished using inactivated vaccines, most of which do not include adjuvants [92]. The CDC estimates that, for the especially severe 2024–2025 influenza season, influenza vaccination prevented 10 million symptomatic illnesses, up to 180,000 hospitalizations, and up to 12,000 deaths [93].

Influenza virus also poses a major economic risk to the agricultural sector. For example, the ongoing outbreak of H5N1 Highly Pathogenic Avian Influenza (HPAI) has been projected to result in a loss of 0.06 to 0.9% of the US gross domestic product [94]. Pigs are highly susceptible to influenza virus strains originating not only in swine but also in humans and birds [95]. Clinical signs observed in pigs infected with influenza A virus, including lethargy, appetite loss, coughing, and nasal discharge, rarely cause death but often result in weight loss, affecting productivity and reproductive efficiency [96]. As in humans, inactivated vaccines are the primary approach used to control influenza virus in swine; however, unlike influenza vaccination in humans, most inactivated swine influenza vaccines are adjuvanted with oil and/or alum [95].

### 7.1. Clinical and Pathological Findings

A series of suspected IAV-associated VAERD cases was identified in swine in northwest Germany given commercial inactivated IAV vaccines [97]. Following sudden death with no premonitory signs, the six sows that underwent a necropsy were all found to have necrotizing bronchopneumonia. Compared to healthy control pigs and younger pigs infected with IAV or with *Actinobacillus pleuropneumoniae*, lungs of the sick sows demonstrated a significant increase in the number of eosinophilic peroxidase (EPO)-positive eosinophils. Other lung inflammatory infiltrates included CD3+ T cells, CD20+ B cells, and IBA1+ macrophages [97], similar to inflammation seen in the lungs of FI-RSV-vaccinated children with VAERD [5]. While no IAV antigen was found, IAV RNA, which was typed as avian-origin HA in two samples, was detected in all six sows, leading the authors to conclude the sows were suffering from VAERD induced by a breakthrough IAV infection [97]. While not definitive, these cases were consistent with the clinical and pathological features of VAERD and thus represent the first known field cases of IAV-associated VAERD in a livestock species. However, further investigation into the specific mechanisms driving IAV-associated VAERD in livestock species is warranted.

### 7.2. Relevance to IAV Vaccines in Humans

Conflicting evidence exists regarding the relevance of immune enhancement of disease involving influenza viruses [3,45]. In preclinical studies involving pigs, ferrets, and mice, researchers observed that non-neutralizing antibodies and pulmonary immune complex deposition, leading to complement activation, were linked to enhanced disease [18,41,42,43,44,98,99,100]. However, unlike inactivated IAV vaccines, we found no reports of IAV vaccines engineered through alternative approaches (e.g., replicon particle vaccine, live-attenuated bat influenza virus vectored vaccine) inducing VAERD in pigs following heterologous challenge [101,102].

Influenza vaccines used in humans have a long and robust safety record, and it is unclear whether the results from animal studies have any bearing on influenza vaccination in humans. Human influenza vaccines are not reported to induce fusion-promoting antibodies or ADE, and clinical studies do not report enhanced disease associated with monoclonal antibody therapies. Moreover, targeting the HA stem region, to which fusion-promoting antibodies bind, is not predictive of disease enhancement, as antibodies to this region can also be protective [3]. In humans, a limited number of studies noted a correlation between prior vaccination with the 2008–2009 seasonal influenza vaccine and increased risk of severe disease during the 2009 H1N1 pandemic [33,103], with evidence of pulmonary immune complex deposition and complement activation as well as robust T cell infiltrates in autopsy specimens from deceased patients [3]. The same group reported that cross-reactive, poorly neutralizing antibodies were associated with an enhanced risk of disease in middle-aged patients during the 2009 H1N1 pandemic [33]. Importantly, other epidemiological studies found no association between prior immune status and increased disease during the 2009 H1N1 pandemic, and many reported that preexisting immunity was protective [104,105,106]. Additionally, widespread surveillance of seasonal influenza infections and seasonal influenza vaccine safety and efficacy has not reported VAERD or other immune enhancement, despite regular antigenic drift and consequently reduced protection conferred by prior exposure [3]. Therefore, while VAERD can occur in animal models and veterinary settings, little evidence suggests it is an issue in human influenza vaccination.

## 8. Comparison Across Viruses

The relative weight given to these mechanisms differs by virus, reflecting both genuine biological differences and differences in the evidence available to investigators.

For RSV and IAV, humoral and immune complex-mediated mechanisms feature prominently in part because human material could be examined directly: post-mortem specimens from the FI-RSV trials and from fatal 2009 H1N1 cases were available for immunohistochemical analysis and demonstrated complement deposition [32,33]. Influenza further offers a defined structural mechanism by which non-neutralizing antibodies can enhance infection [44], and decades of seasonal vaccination provide human epidemiological data against which enhancement hypotheses can be tested. Both viruses also cause disease in immunologically naïve infants or in hosts with complex prior antigenic exposure, contexts in which antibody quality is particularly consequential.

For coronaviruses, by contrast, nearly all evidence comes from vaccine–challenge experiments in laboratory animals, with primary readouts of pulmonary histopathology and pulmonary cellular or cytokine profiles. Eosinophil infiltration and type 2 cytokine production were the endpoints most consistently measured and, correspondingly, the findings most consistently reported [2,74,88]. The apparent predominance of cellular mechanisms in coronavirus VAERD should therefore be interpreted with the recognition that it reflects, in part, what has been measured and in which experimental systems, rather than an established biological distinction between viral families. Where investigators have specifically sought antibody-driven mechanisms in coronavirus models, they have found evidence for them, as in the MERS-CoV rabbit studies described above [34]. This interpretive limitation aligns with the broader critique of methodological heterogeneity in this field, also discussed in this review [2,89,90].

Notwithstanding these differences, a common set of features recurs across the examples reviewed here: (1) an antigen that has lost or does not display protective conformational epitopes; (2) an antibody response that is abundant but poorly neutralizing; (3) non-replicating delivery that may provide limited innate stimulation and consequently poor affinity maturation; (4) an antigen-inexperienced or immunologically immature host; (5) a CD4-dominant, type 2-skewed memory response with weak cytotoxic and regulatory components; and (6) subsequent exposure to a virus that the primed response cannot control. Adjuvant selection, antigen dose, route, and interval modify these variables but have not been shown to act as independent determinants of enhancement.

Finally, the presence of these mechanisms is not itself equivalent to VAERD. Type 2 inflammation, eosinophil recruitment, and complement deposition all occur during natural RSV and influenza infection in unvaccinated hosts [22,63,66], and each may be detected in vaccinated animals that nonetheless experience disease equivalent to or milder than that of unvaccinated controls. The key determinant of whether VAERD is occurring is whether vaccinated individuals or animals develop more severe clinical disease and/or pulmonary pathology or inflammation following infection than their unvaccinated, infected counterparts.

## 9. Strategies for Avoiding VAERD Risk

Based on results from animal systems and experience with inactivated RSV vaccination, vaccines that fail to elicit robust protective immunity, often in combination with T_H_2-biased immune responses, are most commonly associated with VAERD. Therefore, correlates of immune protection for the target pathogen should be carefully considered during vaccine design, and vaccine antigens should be carefully assessed to ensure that immune epitopes associated with protective immunity are preserved during vaccine production. Where possible, it is also important to subject the vaccine and any associated adjuvants to extensive testing in animal models of disease, with careful attention paid to any safety signals that might indicate enhanced respiratory pathology or viral loads in vaccinated individuals upon pathogen challenge. Likewise, if antigenic variation is an issue in the target respiratory pathogen, the vaccine should, where possible, be tested against these variants to ensure that breakthrough infection associated with loss of protective immunity does not result in enhanced disease. Lastly, as evidenced by experience with the inactivated RSV vaccine, careful monitoring for safety signals during clinical trials is essential to ensure that newly developed vaccines are safe and effective in human populations.

## 10. Considerations for Clinical Practice

From a clinical standpoint, the most important observation about VAERD is that it is not presently a diagnosis that can be made at the bedside. No validated clinical criteria distinguish VAERD from severe respiratory viral illness occurring in a vaccinated patient, and no routine laboratory or imaging finding makes that distinction. The case definition developed by the Coalition for Epidemic Preparedness Innovations was designed for clinical trial and surveillance settings and relies on comparisons between vaccinated and unvaccinated groups rather than on assessment of the individual patient [2]. Because severe respiratory infection in vaccinated individuals is common while VAERD is exceptionally rare, encouraging clinicians to include it in the differential diagnosis of severe respiratory illness would likely generate far more misattribution than detection and risk eroding confidence in vaccines with demonstrated benefit. The clinician’s contribution is better located in the systems that do possess the power to detect enhancement, several of which depend directly on practice at the point of care.

### 10.1. Documentation and Reporting

Detecting vaccine-associated adverse outcomes requires accurate records of vaccination status, product, and timing relative to symptom onset. Where these are absent or incomplete in patients hospitalized with severe respiratory infection, epidemiological analysis is correspondingly weakened. Consistent use of immunization information systems and structured documentation of vaccination history are low-cost contributions to a capability that is difficult to reconstruct retrospectively. Passive surveillance systems such as the Vaccine Adverse Event Reporting System in the United States, and comparable national systems elsewhere, depend on clinician reporting of unexpected outcomes. Reports need not establish causation, and clinicians should not be deterred from reporting by uncertainty as to whether a given case represents enhancement, vaccine failure, or coincidental severe infection; distinguishing among these is the function of the surveillance system rather than a prerequisite for reporting to it.

### 10.2. Retention of Tissue and Specimens

Nearly everything known about VAERD mechanisms in humans comes from post-mortem material. Reanalysis of specimens from the two children who died, conducted more than three decades after their deaths, demonstrated peribronchiolar C4d deposition and established a role for immune complexes in FI-RSV VAERD [32]; immune complex findings in pandemic H1N1 influenza came from autopsy lung specimens [33]. Pursuit of autopsy in unexplained deaths from severe respiratory infection, retention of appropriately fixed tissue, and banking of acute and convalescent sera preserves the capacity to perform mechanistic investigations in the future if they become necessary.

### 10.3. Trial Safety Monitoring

Clinicians involved in early-phase trials of vaccines against respiratory viruses, particularly those using novel antigens, platforms, or adjuvants, are well positioned to address enhancement prospectively rather than retrospectively. This includes prespecified endpoints capturing the severity of breakthrough infection in vaccinated relative to control participants and attention to populations in which the preclinical literature suggests theoretical risk may be greater, such as antigen-naïve infants.

### 10.4. Communication

Clinicians are frequently asked about vaccine safety, and the FI-RSV trials are periodically invoked in public discussion in ways that overstate their relevance to currently licensed products. An accurate account of this history is more reassuring than a defensive one. Enhanced disease from FI-RSV and FI-MV was real and caused harm; the vaccinology field identified it, spent decades investigating it, and applied what it learned judiciously. Recognizing that inactivation destroyed the conformational epitopes required for protective immunity contributed to the development of structure-guided antigen design, which underlies both current RSV immunization strategies and several COVID-19 vaccines [68,107,108,109]. Relevant contemporary evidence shows that enhancement has been sought but not found in licensed human respiratory vaccines. Clinicians able to convey both halves of that history—that the risk was taken seriously, and that it was addressed—are better placed to sustain confidence than those able only to assert safety.

## 11. Conclusions

VAERD is a rare phenomenon documented in a small number of well-defined settings: the FI-RSV clinical trials of the 1960s, a recent series of field cases in commercially vaccinated swine, and a subset of vaccine–challenge experiments in laboratory animals. Among the reports surveyed here, whole inactivated virus-based vaccines that fail to elicit protective antibody responses and/or induce T_H_2-biased immune responses appear to be most commonly associated with VAERD. The evidence reviewed does not, however, establish these vaccines as independent risk factors. Enhancement in these models depends jointly on antigen quality, vaccine platform, host immune status, challenge strain and dose, and experimental design. Furthermore, although alum-adjuvanted vaccines are associated with VAERD in some instances, alum-adjuvanted formulations have been associated with improved neutralizing responses and protection in other settings. Association within particular experimental systems should not be interpreted as causation, and findings from selected animal models should not be extrapolated to licensed human vaccines.

It is equally important to state what the evidence does not show. VAERD has not been identified in association with any currently licensed human coronavirus vaccine, despite the administration of billions of doses under intensive global pharmacovigilance. Nor has it been identified through decades of seasonal influenza surveillance, despite regular antigenic drift and the variable vaccine effectiveness that follows. Notwithstanding the FI-RSV and FI-MV, both withdrawn during clinical trials or from the market, respectively, more than half a century ago, no licensed human vaccine has been shown to cause enhanced respiratory disease. Vaccination against respiratory viruses prevents a very large burden of illness, hospitalization, and death. That benefit should not be obscured by discussion of a rare and largely experimental adverse outcome.

The practical value of the VAERD literature therefore lies upstream of clinical practice. VAERD is best understood as a defined component of preclinical vaccine safety evaluation, informing the selection of animal models and endpoints, verification of antigenic integrity and conformational quality during manufacture, rational adjuvant selection, the design of safety monitoring in early-phase clinical trials, and post-marketing surveillance. Framed this way, the history recounted in this review is largely a record of success. The failure of FI-RSV was identified during clinical trials and investigated for decades, its mechanisms were substantially resolved, and the resulting understanding of conformation-dependent antibody functionality contributed directly to the structure-guided vaccine design underlying current RSV immunization strategies and several successful and enormously impactful COVID-19 vaccines [68,107,108,109]. Continued attention to VAERD in vaccine development is warranted not because vaccines against respiratory viruses are unsafe, but because systematically evaluating risks like this is part of why they are safe.

## Figures and Tables

**Table 1:** Summary of reports describing exacerbation of clinical disease and/or pathology in vaccinated animals compared with unvaccinated animals following coronavirus infection.

Paper	Animal Model	Coronavirus	Vaccine Approach(es)	Adjuvant	Clinical Disease	Pathology	Viral Replication	Vaccine Protection	Reference
Agrawal et al., 2016	hDPP4 mouse	MERS-CoV	Inactivated	Alum, MF59	N.D.	Increased lung pathology scores and eosinophil infiltration; increased IL-5 and IL-13	N.A.E.	Induction of neutralizing antibodies	[69]
Bolles & Deming et al., 2011	BALB/c mouse	SARS-CoV-1	Inactivated; VRP expressing N protein	Alum	N.A.E. on body weight or survival	Increased lung eosinophil infiltration, increased perivascular and peri-bronchial cuffing	N.A.E.	Poor protection following heterologous viral challenge in aged mice	[70]
Czub & Weingartl et al., 2005	Ferret	SARS-CoV-1	Vaccina virus-vectored S or N proteins		N.A.E. on body temperature or behavior	Periportal and pan-lobular hepatitis; increased liver cell necrosis	N.A.E.	No enhanced viral protection despite development of neutralizing antibodies	[71]
Deming et al., 2006	BALB/c mouse	SARS-CoV-1	VRP expressing N protein		N.D.	Bronchiolitis, alveolitis, and eosinophil infiltration in lungs of VRP-N vaccinated mice	N.A.E.	No homologous or heterologous protection in VRP-N vaccinated mice	[72]
Dillard & Taft-Benz et al., 2024	BALB/c mouse	SARS-CoV-2	Inactivated	Alum	Impaired respiratory function by WBP	Increased lung pathology scores and eosinophil infiltration	Delayed viral clearance	Complete homologous protection, incomplete heterologous protection	[73]
DiPiazza & Leist et al., 2021	BALB/c mouse	SARS-CoV-2	Inactivated; Denatured S protein	Alum	N.A.E. on body weight	Increased lung inflammation, pathology, and eosinophil infiltration; Type II pneumocyte hyperplasia; elevated IL-4 and IL-5 in lung	N.A.E.	Suboptimal neutralizing antibody potency	[74]
Ebenig et al., 2022	Syrian hamster	SARS-CoV-2	Non-stabilized S protein	Alum	Increased weight loss	Increased inflammation in vascular endothelium and infiltration of eosinophils in lungs	N.A.E.	Poor protection and induction of neutralizing antibodies	[75]
Hashem & Algaissi et al., 2019	hDDP4 mouse	MERS-CoV	Adenovirus-vectored S1 domain		N.A.E. on body weight or survival	Pulmonary perivascular hemorrhage	N.A.E.	Strong protection against lethal infection	[76]
Honda-Okubo et al., 2015	BALB/c mouse	SARS-CoV-1	Recombinant S protein; Inactivated	Alum; unadjuvanted	N.A.E. on survival	Pulmonary eosinophilia	N.A.E.	Strong protection against mortality; poor protection against infection following unadjuvanted vaccination	[77]
Iwata-Yoshikawa et al., 2014	BALB/c mouse	SARS-CoV-1	Inactivated	Alum; unadjuvanted	N.A.E. on body weight or survival	Pulmonary eosinophilia	N.A.E.	Partial protection against lethal disease	[78]
Iwata-Yoshikawa et al., 2022	BALB/c mouse	SARS-CoV-2	Recombinant S protein ectodomain	Alum; unadjuvanted	N.A.E. on body weight or survival	Pulmonary eosinophilia	N.A.E.	Low neutralizing antibody titers; only partial protection against lethal infected in unadjuvanted vaccinated mice	[79]
Liu et al., 2019	Rhesus macaque	SARS-CoV-1	Vaccina virus-vectored S protein		N.A.E. on body temperature	Increased lung histopathology scores	N.A.E.	Strong protection against infection; poor protection against clinical disease development (fever)	[80]
Menachery et al., 2015	BALB/c mouse	SARS-CoV-1	Inactivated	Alum	Increased weight loss	Increased lung histopathology and eosinophil scores	N.A.E.	Poor protection from heterologous CoV infection	[81]
Menachery et al., 2016	BALB/c mouse	SARS-CoV-1	Inactivated	Alum	N.A.E. on body weight	Increased lung eosinophil scores	N.A.E.	Poor protection from heterologous CoV infection	[82]
Tseng et al., 2012	BALB/c and C57BL/6 mice	SARS-CoV-1	Inactivated; S protein subunit	Alum; unadjuvanted	N.D.	Increased pulmonary eosinophil infiltration	N.A.E.	Strong induction of neutralizing antibodies and protection from infection	[83]
Wang, Zhang, & Kuwahara et al., 2016	Rhesus macaque	SARS-CoV-1	Inactivated; S peptide epitope mixture		N.D.	Subjectively worsened lung pathology in one inactivated vaccine animal and one peptide epitope group	N.A.E.	Inadequate protection against lung pathology in one inactivated vaccine macaque and one peptide epitope group	[84]
Weingartl and Czub et al., 2004	Ferret	SARS-CoV-1	Vaccina virus-vectored S protein		N.A.E. on body temperature or behavior	Increased serum ALT levels of alanine aminotransferase; periportal and pan-lobular mononuclear hepatitis with cellular necrosis	N.A.E.	Strong induction of neutralizing antibodies	[85]
Yasui et al., 2008	BALB/c mouse	SARS-CoV-1	Vaccina virus-vectored N protein		N.D.	Increased pathology scores, immune cell infiltration, and type 2-associated cytokine gene expression in lungs	N.A.E.	Poor protection from infection	[86]
Zhang et al., 2024	K18-hACE2 mouse	SARS-CoV-2	Recombinant S protein ectodomain	Alum + CpG	N.A.E. on body weight or survival	Increased pulmonary basophil, eosinophil infiltration and peri-bronchial inflammation; increased T_H_2- and T_H_17 cells and cytokine gene expression in lungs	N.A.E.	Partial protection against weight loss, mortality, and infection	[87]

N.D. = no data; N.A.E. = no adverse effects; VRP = viral replicon particle; N protein = nucleocapsid protein; S protein/peptide = Spike protein/peptide; WBP = whole body plethysmography.

## Data Availability

No new data were created or analyzed in this study. Data sharing is not applicable to this article.

## References

[1] Haynes B.F., Corey L., Fernandes P., Gilbert P.B., Hotez P.J., Rao S., Santos M.R., Schuitemaker H., Watson M., Arvin A. (2020). Prospects for a Safe COVID-19 Vaccine. Sci. Transl. Med..

[2] Munoz F.M., Cramer J.P., Dekker C.L., Dudley M.Z., Graham B.S., Gurwith M., Law B., Perlman S., Polack F.P., Spergel J.M. (2021). Vaccine-Associated Enhanced Disease: Case Definition and Guidelines for Data Collection, Analysis, and Presentation of Immunization Safety Data. Vaccine.

[3] Arvin A.M., Fink K., Schmid M.A., Cathcart A., Spreafico R., Havenar-Daughton C., Lanzavecchia A., Corti D., Virgin H.W. (2020). A Perspective on Potential Antibody-Dependent Enhancement of SARS-CoV-2. Nature.

[4] Polack F.P. (2007). Atypical Measles and Enhanced Respiratory Syncytial Virus Disease (ERD) Made Simple. Pediatr. Res..

[5] Ruckwardt T.J., Morabito K.M., Graham B.S. (2019). Immunological Lessons from Respiratory Syncytial Virus Vaccine Development. Immunity.

[6] Killikelly A.M., Kanekiyo M., Graham B.S. (2016). Pre-Fusion F Is Absent on the Surface of Formalin-Inactivated Respiratory Syncytial Virus. Sci. Rep..

[7] Kim H.W., Canchola J.G., Brandt C.D., Pyles G., Chanock R.M., Jensen K., Parrott R.H. (1969). Respiratory Syncytial Virus Disease in Infants Despite Prior Administration of Antigenic Inactivated Vaccine. Am. J. Epidemiol..

[8] Murphy B.R., Walsh E.E. (1988). Formalin-Inactivated Respiratory Syncytial Virus Vaccine Induces Antibodies to the Fusion Glycoprotein That Are Deficient in Fusion-Inhibiting Activity. J. Clin. Microbiol..

[9] Chin J., Magoffin R.L., Shearer L.A., Schieble J.H., Lennette E.H. (1969). Field Evaluation of a Respiratory Syncytial Virus Vaccine and Trivalent Parainfluenza Virus Vaccine in a Pediatric Population. Am. J. Epidemiol..

[10] Filginiti V.A., Eller J.J., Sieber O.F., Joyner J.W., Minamitani M., Meiklejohn G. (1969). Respiratory Virus Immunization. I. A Field Trial of Two Inactivated Respiratory Virus Vaccines; an Aqueous Trivalent Parainfluenza Virus Vaccine and an Alum-Precipitated Respiratory Syncytial Virus Vaccine. Am. J. Epidemiol..

[11] Mitchell R.H., Kapikian A.Z., Chanock R.M., Shvedoff R.A., Stewart C.E. (1969). An Epidemiologic Study of Altered Clinical Reactivity to Respiratory Syncytial (RS) Virus Infection in Children Previously Vaccinated with an Inactivated RS Virus Vaccine. Am. J. Epidemiol..

[12] Delgado M.F., Coviello S., Monsalvo A.C., Melendi G.A., Hernandez J.Z., Batalle J.P., Diaz L., Trento A., Chang H.-Y., Mitzner W. (2009). Lack of Antibody Affinity Maturation Due to Poor Toll-like Receptor Stimulation Leads to Enhanced Respiratory Syncytial Virus Disease. Nat. Med..

[13] Polack F.P., Hoffman S.J., Crujeiras G., Griffin D.E. (2003). A Role for Nonprotective Complement-Fixing Antibodies with Low Avidity for Measles Virus in Atypical Measles. Nat. Med..

[14] Johnson T.R., Rao S., Seder R.A., Chen M., Graham B.S. (2009). TLR9 Agonist, but Not TLR7/8, Functions as an Adjuvant to Diminish FI-RSV Vaccine-Enhanced Disease, While Either Agonist Used as Therapy during Primary RSV Infection Increases Disease Severity. Vaccine.

[15] Delgado M.F., Polack F.P. (2004). Involvement of Antibody, Complement and Cellular Immunity in the Pathogenesis of Enhanced Respiratory Syncytial Virus Disease. Expert Rev. Vaccines.

[16] Acosta P.L., Caballero M.T., Polack F.P. (2015). Brief History and Characterization of Enhanced Respiratory Syncytial Virus Disease. Clin. Vaccine Immunol..

[17] Openshaw P.J.M., Chiu C., Culley F.J., Johansson C. (2017). Protective and Harmful Immunity to RSV Infection. Annu. Rev. Immunol..

[18] Rajao D.S., Sandbulte M.R., Gauger P.C., Kitikoon P., Platt R., Roth J.A., Perez D.R., Loving C.L., Vincent A.L. (2016). Heterologous Challenge in the Presence of Maternally-Derived Antibodies Results in Vaccine-Associated Enhanced Respiratory Disease in Weaned Piglets. Virology.

[19] Durbin J.E., Durbin R.K. (2004). Respiratory Syncytial Virus-Induced Immunoprotection and Immunopathology. Viral Immunol..

[20] Eichinger K.M., Kosanovich J.L., Gidwani S.V., Zomback A., Lipp M.A., Perkins T.N., Oury T.D., Petrovsky N., Marshall C.P., Yondola M.A. (2020). Prefusion RSV F Immunization Elicits Th2-Mediated Lung Pathology in Mice When Formulated with a Th2 (but Not a Th1/Th2-Balanced) Adjuvant Despite Complete Viral Protection. Front. Immunol..

[21] Kosanovich J.L., Eichinger K.M., Lipp M.A., Yondola M.A., Perkins T.N., Empey K.M. (2020). Formulation of the Prefusion RSV F Protein with a Th1/Th2-Balanced Adjuvant Provides Complete Protection without Th2-Skewed Immunity in RSV-Experienced Young Mice. Vaccine.

[22] Castilow E.M., Olson M.R., Varga S.M. (2007). Understanding Respiratory Syncytial Virus (RSV) Vaccine-Enhanced Disease. Immunol. Res..

[23] Graham B.S., Henderson G.S., Tang Y.W., Lu X., Neuzil K.M., Colley D.G. (1993). Priming Immunization Determines T Helper Cytokine mRNA Expression Patterns in Lungs of Mice Challenged with Respiratory Syncytial Virus. J. Immunol..

[24] Kalina W.V., Woolums A.R., Berghaus R.D., Gershwin L.J. (2004). Formalin-Inactivated Bovine RSV Vaccine Enhances a Th2 Mediated Immune Response in Infected Cattle. Vaccine.

[25] Kalina W.V., Woolums A.R., Gershwin L.J. (2005). Formalin-Inactivated Bovine RSV Vaccine Influences Antibody Levels in Bronchoalveolar Lavage Fluid and Disease Outcome in Experimentally Infected Calves. Vaccine.

[26] Moghaddam A., Olszewska W., Wang B., Tregoning J.S., Helson R., Sattentau Q.J., Openshaw P.J.M. (2006). A Potential Molecular Mechanism for Hypersensitivity Caused by Formalin-Inactivated Vaccines. Nat. Med..

[27] de Swart R.L., Kuiken T., Timmerman H.H., van Amerongen G., van den Hoogen B.G., Vos H.W., Neijens H.J., Andeweg A.C., Osterhaus A.D.M.E. (2002). Immunization of Macaques with Formalin-Inactivated Respiratory Syncytial Virus (RSV) Induces Interleukin-13-Associated Hypersensitivity to Subsequent RSV Infection. J. Virol..

[28] Waris M.E., Tsou C., Erdman D.D., Zaki S.R., Anderson L.J. (1996). Respiratory Synctial Virus Infection in BALB/c Mice Previously Immunized with Formalin-Inactivated Virus Induces Enhanced Pulmonary Inflammatory Response with a Predominant Th2-like Cytokine Pattern. J. Virol..

[29] Connors M., Kulkarni A.B., Firestone C.Y., Holmes K.L., Morse H.C., Sotnikov A.V., Murphy B.R. (1992). Pulmonary Histopathology Induced by Respiratory Syncytial Virus (RSV) Challenge of Formalin-Inactivated RSV-Immunized BALB/c Mice Is Abrogated by Depletion of CD4+ T Cells. J. Virol..

[30] Connors M., Giese N.A., Kulkarni A.B., Firestone C.Y., Morse H.C., Murphy B.R. (1994). Enhanced Pulmonary Histopathology Induced by Respiratory Syncytial Virus (RSV) Challenge of Formalin-Inactivated RSV-Immunized BALB/c Mice Is Abrogated by Depletion of Interleukin-4 (IL-4) and IL-10. J. Virol..

[31] Knudson C.J., Hartwig S.M., Meyerholz D.K., Varga S.M. (2015). RSV Vaccine-Enhanced Disease Is Orchestrated by the Combined Actions of Distinct CD4 T Cell Subsets. PLoS Pathog..

[32] Polack F.P., Teng M.N., L.Collins P., Prince G.A., Exner M., Regele H., Lirman D.D., Rabold R., Hoffman S.J., Karp C.L. (2002). A Role for Immune Complexes in Enhanced Respiratory Syncytial Virus Disease. J. Exp. Med..

[33] Monsalvo A.C., Batalle J.P., Lopez M.F., Krause J.C., Klemenc J., Hernandez J.Z., Maskin B., Bugna J., Rubinstein C., Aguilar L. (2011). Severe Pandemic 2009 H1N1 Influenza Disease Due to Pathogenic Immune Complexes. Nat. Med..

[34] Houser K.V., Broadbent A.J., Gretebeck L., Vogel L., Lamirande E.W., Sutton T., Bock K.W., Minai M., Orandle M., Moore I.N. (2017). Enhanced Inflammation in New Zealand White Rabbits When MERS-CoV Reinfection Occurs in the Absence of Neutralizing Antibody. PLoS Pathog..

[35] Jeyanathan M., Afkhami S., Smaill F., Miller M.S., Lichty B.D., Xing Z. (2020). Immunological Considerations for COVID-19 Vaccine Strategies. Nat. Rev. Immunol..

[36] Katzelnick L.C., Gresh L., Halloran M.E., Mercado J.C., Kuan G., Gordon A., Balmaseda A., Harris E. (2017). Antibody-Dependent Enhancement of Severe Dengue Disease in Humans. Science.

[37] Murray S.M., Ansari A.M., Frater J., Klenerman P., Dunachie S., Barnes E., Ogbe A. (2023). The Impact of Pre-Existing Cross-Reactive Immunity on SARS-CoV-2 Infection and Vaccine Responses. Nat. Rev. Immunol..

[38] Pulendran B., Ahmed R. (2011). Immunological Mechanisms of Vaccination. Nat. Immunol..

[39] Tekes G., Thiel H.-J. (2016). Chapter Six Feline Coronaviruses Pathogenesis of Feline Infectious Peritonitis. Adv. Virus Res..

[40] Tizard I.R. (2020). Vaccination against Coronaviruses in Domestic Animals. Vaccine.

[41] Khurana S., Loving C.L., Manischewitz J., King L.R., Gauger P.C., Henningson J., Vincent A.L., Golding H. (2013). Vaccine-Induced Anti-HA2 Antibodies Promote Virus Fusion and Enhance Influenza Virus Respiratory Disease. Sci. Transl. Med..

[42] Kimble J.B., Brand M.W., Kaplan B.S., Gauger P., Coyle E.M., Chilcote K., Khurana S., Vincent A.L. (2022). Vaccine-Associated Enhanced Respiratory Disease Following Influenza Virus Infection in Ferrets Recapitulates the Model in Pigs. J. Virol..

[43] Smatti M.K., Thani A.A.A., Yassine H.M. (2018). Viral-Induced Enhanced Disease Illness. Front. Microbiol..

[44] Winarski K.L., Tang J., Klenow L., Lee J., Coyle E.M., Manischewitz J., Turner H.L., Takeda K., Ward A.B., Golding H. (2019). Antibody-Dependent Enhancement of Influenza Disease Promoted by Increase in Hemagglutinin Stem Flexibility and Virus Fusion Kinetics. Proc. Natl. Acad. Sci. USA.

[45] Bournazos S., Gupta A., Ravetch J.V. (2020). The Role of IgG Fc Receptors in Antibody-Dependent Enhancement. Nat. Rev. Immunol..

[46] Kruijsen D., Schijf M.A., Lukens M.V., van Uden N.O., Kimpen J.L., Coenjaerts F.E., Bleek G.M. (2011). van Local Innate and Adaptive Immune Responses Regulate Inflammatory Cell Influx into the Lungs after Vaccination with Formalin Inactivated RSV. Vaccine.

[47] Olson M.R., Varga S.M. (2007). CD8 T Cells Inhibit Respiratory Syncytial Virus (RSV) Vaccine-Enhanced Disease. J. Immunol..

[48] Waris M.E., Tsou C., Erdman D.D., Day D.B., Anderson L.J. (1997). Priming with Live Respiratory Syncytial Virus (RSV) Prevents the Enhanced Pulmonary Inflammatory Response Seen after RSV Challenge in BALB/c Mice Immunized with Formalin-Inactivated RSV. J. Virol..

[49] Loebbermann J., Durant L., Thornton H., Johansson C., Openshaw P.J. (2013). Defective Immunoregulation in RSV Vaccine-Augmented Viral Lung Disease Restored by Selective Chemoattraction of Regulatory T Cells. Proc. Natl. Acad. Sci. USA.

[50] Srikiatkhachorn A., Braciale T.J. (1997). Virus-Specific CD8+ T Lymphocytes Downregulate T Helper Cell Type 2 Cytokine Secretion and Pulmonary Eosinophilia during Experimental Murine Respiratory Syncytial Virus Infection. J. Exp. Med..

[51] Halstead S.B., Chow J.S., Marchette N.J. (1973). Immunological Enhancement of Dengue Virus Replication. Nat. New Biol..

[52] Halstead S.B. (2017). Dengvaxia Sensitizes Seronegatives to Vaccine Enhanced Disease Regardless of Age. Vaccine.

[53] Jiang J., Ramos S.J., Bangalore P., Elwood D., Cashman K.A., Kudchodkar S.B., Schultheis K., Pugh H., Walters J., Tur J. (2021). Multivalent DNA Vaccines as a Strategy to Combat Multiple Concurrent Epidemics: Mosquito-Borne and Hemorrhagic Fever Viruses. Viruses.

[54] Sridhar S., Luedtke A., Langevin E., Zhu M., Bonaparte M., Machabert T., Savarino S., Zambrano B., Moureau A., Khromava A. (2018). Effect of Dengue Serostatus on Dengue Vaccine Safety and Efficacy. N. Engl. J. Med..

[55] Fulginiti V.A., Eller J.J., Downie A.W., Kempe C.H. (1967). Altered Reactivity to Measles Virus: Atypical Measles in Children Previously Immunized with Inactivated Measles Virus Vaccines. JAMA.

[56] Martin D.B., Weiner L.B., Nieburg P.I., Blair D.C. (1979). Atypical Measles in Adolescents and Young Adults. Ann. Intern. Med..

[57] Rauh L.W., Schmidt R. (1965). Measles Immunization with Killed Virus Vaccine: Serum Antibody Titers and Experience with Exposure to Measles Epidemic. Am. J. Dis. Child..

[58] Young L.W., Smith D.I., Glasgow L.A. (1970). Pneumonia of Atypical Measles: Residual Nodular Lesions. Am. J. Roentgenol. Radium Ther. Nucl. Med..

[59] Scott F.W. (1987). Coronaviruses. Adv. Exp. Med. Biol..

[60] Addie D.D., Paltrinieri S., Pedersen N.C., Second International Feline Coronavirus/Feline Infectious Peritonitis Symposium (2004). Recommendations from Workshops of the Second International Feline Coronavirus/Feline Infectious Peritonitis Symposium. J. Feline Med. Surg..

[61] Li Y., Wang X., Blau D.M., Caballero M.T., Feikin D.R., Gill C.J., Madhi S.A., Omer S.B., Simões E.A.F., Campbell H. (2022). Global, Regional, and National Disease Burden Estimates of Acute Lower Respiratory Infections Due to Respiratory Syncytial Virus in Children Younger than 5 Years in 2019: A Systematic Analysis. Lancet.

[62] Mejias A., Rodríguez-Fernández R., Oliva S., Peeples M.E., Ramilo O. (2020). The Journey to a Respiratory Syncytial Virus Vaccine. Ann. Allergy Asthma Immunol..

[63] Openshaw P.J.M., Culley F.J., Olszewska W. (2001). Immunopathogenesis of Vaccine-Enhanced RSV Disease. Vaccine.

[64] Mazur N.I., Higgins D., Nunes M.C., Melero J.A., Langedijk A.C., Horsley N., Buchholz U.J., Openshaw P.J., McLellan J.S., Englund J.A. (2018). The Respiratory Syncytial Virus Vaccine Landscape: Lessons from the Graveyard and Promising Candidates. Lancet Infect. Dis..

[65] Prince G.A., Curtis S.J., Yim K.C., Porter D.D. (2001). Vaccine-Enhanced Respiratory Syncytial Virus Disease in Cotton Rats Following Immunization with Lot 100 or a Newly Prepared Reference Vaccine. J. Gen. Virol..

[66] Openshaw P.J.M., Tregoning J.S. (2005). Immune Responses and Disease Enhancement during Respiratory Syncytial Virus Infection. Clin. Microbiol. Rev..

[67] Muralidharan A., Li C., Wang L., Li X. (2017). Immunopathogenesis Associated with Formaldehyde-Inactivated RSV Vaccine in Preclinical and Clinical Studies. Expert Rev. Vaccines.

[68] Graham B.S. (2020). Rapid COVID-19 Vaccine Development. Science.

[69] Agrawal A.S., Tao X., Algaissi A., Garron T., Narayanan K., Peng B.-H., Couch R.B., Tseng C.-T.K. (2016). Immunization with Inactivated Middle East Respiratory Syndrome Coronavirus Vaccine Leads to Lung Immunopathology on Challenge with Live Virus. Hum. Vaccines Immunother..

[70] Bolles M., Deming D., Long K., Agnihothram S., Whitmore A., Ferris M., Funkhouser W., Gralinski L., Totura A., Heise M. (2011). A Double-Inactivated Severe Acute Respiratory Syndrome Coronavirus Vaccine Provides Incomplete Protection in Mice and Induces Increased Eosinophilic Proinflammatory Pulmonary Response upon Challenge. J. Virol..

[71] Czub M., Weingartl H., Czub S., He R., Cao J. (2005). Evaluation of Modified Vaccinia Virus Ankara Based Recombinant SARS Vaccine in Ferrets. Vaccine.

[72] Deming D., Sheahan T., Heise M., Yount B., Davis N., Sims A., Suthar M., Harkema J., Whitmore A., Pickles R. (2006). Vaccine Efficacy in Senescent Mice Challenged with Recombinant SARS-CoV Bearing Epidemic and Zoonotic Spike Variants. PLoS Med..

[73] Dillard J.A., Taft-Benz S.A., Knight A.C., Anderson E.J., Pressey K.D., Parotti B., Martinez S.A., Diaz J.L., Sarkar S., Madden E.A. (2024). Adjuvant-Dependent Impact of Inactivated SARS-CoV-2 Vaccines during Heterologous Infection by a SARS-Related Coronavirus. Nat. Commun..

[74] DiPiazza A.T., Leist S.R., Abiona O.M., Moliva J.I., Werner A., Minai M., Nagata B.M., Bock K.W., Phung E., Schäfer A. (2021). COVID-19 Vaccine mRNA-1273 Elicits a Protective Immune Profile in Mice That Is Not Associated with Vaccine-Enhanced Disease upon SARS-CoV-2 Challenge. Immunity.

[75] Ebenig A., Muraleedharan S., Kazmierski J., Todt D., Auste A., Anzaghe M., Gömer A., Postmus D., Gogesch P., Niles M. (2022). Vaccine-Associated Enhanced Respiratory Pathology in COVID-19 Hamsters after TH2-Biased Immunization. Cell Rep..

[76] Hashem A.M., Algaissi A., Agrawal A.S., Al-amri S.S., Alhabbab R.Y., Sohrab S.S., Almasoud A.S., Alharbi N.K., Peng B.-H., Russell M. (2019). A Highly Immunogenic, Protective, and Safe Adenovirus-Based Vaccine Expressing Middle East Respiratory Syndrome Coronavirus S1-CD40L Fusion Protein in a Transgenic Human Dipeptidyl Peptidase 4 Mouse Model. J. Infect. Dis..

[77] Honda-Okubo Y., Barnard D., Ong C.H., Peng B.-H., Tseng C.-T.K., Petrovsky N. (2015). Severe Acute Respiratory Syndrome-Associated Coronavirus Vaccines Formulated with Delta Inulin Adjuvants Provide Enhanced Protection While Ameliorating Lung Eosinophilic Immunopathology. J. Virol..

[78] Iwata-Yoshikawa N., Uda A., Suzuki T., Tsunetsugu-Yokota Y., Sato Y., Morikawa S., Tashiro M., Sata T., Hasegawa H., Nagata N. (2014). Effects of Toll-Like Receptor Stimulation on Eosinophilic Infiltration in Lungs of BALB/c Mice Immunized with UV-Inactivated Severe Acute Respiratory Syndrome-Related Coronavirus Vaccine. J. Virol..

[79] Iwata-Yoshikawa N., Shiwa N., Sekizuka T., Sano K., Ainai A., Hemmi T., Kataoka M., Kuroda M., Hasegawa H., Suzuki T. (2022). A Lethal Mouse Model for Evaluating Vaccine-Associated Enhanced Respiratory Disease during SARS-CoV-2 Infection. Sci. Adv..

[80] Liu L., Wei Q., Lin Q., Fang J., Wang H., Kwok H., Tang H., Nishiura K., Peng J., Tan Z. (2019). Anti–Spike IgG Causes Severe Acute Lung Injury by Skewing Macrophage Responses during Acute SARS-CoV Infection. JCI Insight.

[81] Menachery V.D., Yount B.L., Debbink K., Agnihothram S., Gralinski L.E., Plante J.A., Graham R.L., Scobey T., Ge X.-Y., Donaldson E.F. (2015). A SARS-like Cluster of Circulating Bat Coronaviruses Shows Potential for Human Emergence. Nat. Med..

[82] Menachery V.D., Yount B.L., Sims A.C., Debbink K., Agnihothram S.S., Gralinski L.E., Graham R.L., Scobey T., Plante J.A., Royal S.R. (2016). SARS-like WIV1-CoV Poised for Human Emergence. Proc. Natl. Acad. Sci. USA.

[83] Tseng C.-T., Sbrana E., Iwata-Yoshikawa N., Newman P.C., Garron T., Atmar R.L., Peters C.J., Couch R.B. (2012). Immunization with SARS Coronavirus Vaccines Leads to Pulmonary Immunopathology on Challenge with the SARS Virus. PLoS ONE.

[84] Wang Q., Zhang L., Kuwahara K., Li L., Liu Z., Li T., Zhu H., Liu J., Xu Y., Xie J. (2016). Immunodominant SARS Coronavirus Epitopes in Humans Elicited Both Enhancing and Neutralizing Effects on Infection in Non-Human Primates. ACS Infect. Dis..

[85] Weingartl H., Czub M., Czub S., Neufeld J., Marszal P., Gren J., Smith G., Jones S., Proulx R., Deschambault Y. (2004). Immunization with Modified Vaccinia Virus Ankara-Based Recombinant Vaccine against Severe Acute Respiratory Syndrome Is Associated with Enhanced Hepatitis in Ferrets. J. Virol..

[86] Yasui F., Kai C., Kitabatake M., Inoue S., Yoneda M., Yokochi S., Kase R., Sekiguchi S., Morita K., Hishima T. (2008). Prior Immunization with Severe Acute Respiratory Syndrome (SARS)-Associated Coronavirus (SARS-CoV) Nucleocapsid Protein Causes Severe Pneumonia in Mice Infected with SARS-CoV. J. Immunol..

[87] Zhang T., Magazine N., McGee M.C., Carossino M., Veggiani G., Kousoulas K.G., August A., Huang W. (2024). Th2 and Th17-associated Immunopathology Following SARS-CoV-2 Breakthrough Infection in Spike-vaccinated ACE2-humanized Mice. J. Med. Virol..

[88] Lambert P.-H., Ambrosino D.M., Andersen S.R., Baric R.S., Black S.B., Chen R.T., Dekker C.L., Didierlaurent A.M., Graham B.S., Martin S.D. (2020). Consensus Summary Report for CEPI/BC March 12–13, 2020 Meeting: Assessment of Risk of Disease Enhancement with COVID-19 Vaccines. Vaccine.

[89] Gartlan C., Tipton T., Salguero F.J., Sattentau Q., Gorringe A., Carroll M.W. (2022). Vaccine-Associated Enhanced Disease and Pathogenic Human Coronaviruses. Front. Immunol..

[90] Zellweger R.M., Wartel T.A., Marks F., Song M., Kim J.H. (2020). Vaccination against SARS-CoV-2 and Disease Enhancement—Knowns and Unknowns. Expert Rev. Vaccines.

[91] World Health Organization Up to 650 000 People Die of Respiratory Diseases Linked to Seasonal Flu Each Year. https://www.who.int/news/item/13-12-2017-up-to-650-000-people-die-of-respiratory-diseases-linked-to-seasonal-flu-each-year.

[92] Trombetta C.M., Kistner O., Montomoli E., Viviani S., Marchi S. (2022). Influenza Viruses and Vaccines: The Role of Vaccine Effectiveness Studies for Evaluation of the Benefits of Influenza Vaccines. Vaccines.

[93] Centers for Disease Control and Prevention 2024–2025 Influenza Season Summary: Severity, Disease Burden, and Burden Prevented. https://www.cdc.gov/flu-burden/php/data-vis-vac/2024-2025-prevented.html.

[94] Morel G., Pham A., Morgenstern C., Hicks J.T., Rawson T., Fan V.Y., Edmunds W.J., Forchini G., Hauck K. (2026). An Outbreak of Highly Pathogenic Avian Influenza H5N1 Could Impact the Dairy Cattle Sector and the Broader Economy in the United States. Commun. Earth Environ..

[95] Gracia J.C.M., Pearce D.S., Masic A., Balasch M. (2020). Influenza A Virus in Swine: Epidemiology, Challenges and Vaccination Strategies. Front. Vet. Sci..

[96] Mtaallah O., Germeraad E.A., Puspitarani G.A., Bayle B., Nguyen U.H.P., Meester M., Stegeman A., Apolloni A., Hautefeuille C. (2026). Efficacy of Swine Influenza A Virus Vaccines on Transmission, Viral Shedding and Clinical Signs: Systematic Review and Meta-Analysis. Prev. Vet. Med..

[97] Reineking W., Hennig-Pauka I., Schröder L., Höner U., Schreiber E., Geiping L., Lassnig S., Bonilla M.C., Hewicker-Trautwein M., de Buhr N. (2024). Spontaneous Lethal Outbreak of Influenza A Virus Infection in Vaccinated Sows on Two Farms Suggesting the Occurrence of Vaccine-Associated Enhanced Respiratory Disease with Eosinophilic Lung Pathology. Viruses.

[98] Kobinger G.P., Meunier I., Patel A., Pillet S., Gren J., Stebner S., Leung A., Neufeld J.L., Kobasa D., von Messling V. (2010). Assessment of the Efficacy of Commercially Available and Candidate Vaccines against a Pandemic H1N1 2009 Virus. J. Infect. Dis..

[99] Rajão D.S., Loving C.L., Gauger P.C., Kitikoon P., Vincent A.L. (2014). Influenza A Virus Hemagglutinin Protein Subunit Vaccine Elicits Vaccine-Associated Enhanced Respiratory Disease in Pigs. Vaccine.

[100] Skowronski D.M., Hamelin M.-E., Serres G.D., Janjua N.Z., Li G., Sabaiduc S., Bouhy X., Couture C., Leung A., Kobasa D. (2014). Randomized Controlled Ferret Study to Assess the Direct Impact of 2008–09 Trivalent Inactivated Influenza Vaccine on A(H1N1)Pdm09 Disease Risk. PLoS ONE.

[101] Brand M.W., Anderson T.K., Kitikoon P., Kimble J.B., Otis N., Gauger P.C., Souza C.K., Kaplan B., Mogler M., Strait E. (2022). Bivalent Hemagglutinin and Neuraminidase Influenza Replicon Particle Vaccines Protect Pigs against Influenza a Virus without Causing Vaccine Associated Enhanced Respiratory Disease. Vaccine.

[102] Lee J., Li Y., Li Y., Cino-Ozuna A.G., Duff M., Lang Y., Ma J., Sunwoo S., Richt J.A., Ma W. (2021). Bat Influenza Vectored NS1-Truncated Live Vaccine Protects Pigs against Heterologous Virus Challenge. Vaccine.

[103] Skowronski D.M., Serres G.D., Crowcroft N.S., Janjua N.Z., Boulianne N., Hottes T.S., Rosella L.C., Dickinson J.A., Gilca R., Sethi P. (2010). Association between the 2008–09 Seasonal Influenza Vaccine and Pandemic H1N1 Illness during Spring–Summer 2009: Four Observational Studies from Canada. PLoS Med..

[104] Centers for Disease Control and Prevention (2009). Serum Cross-Reactive Antibody Response to a Novel Influenza A (H1N1) Virus after Vaccination with Seasonal Influenza Vaccine. MMWR Morb. Mortal. Wkly. Rep..

[105] Fisman D.N., Savage R., Gubbay J., Achonu C., Akwar H., Farrell D.J., Crowcroft N.S., Jackson P. (2009). *Older Age and a Reduced Likelihood of* 2009 H1N1 Virus Infection. N. Engl. J. Med..

[106] Mesman A.W., Westerhuis B.M., Ten Hulscher H.I., Jacobi R.H., de Bruin E., van Beek J., Buisman A.M., Koopmans M.P., van Binnendijk R.S. (2016). Influenza Virus A(H1N1)2009 Antibody-Dependent Cellular Cytotoxicity in Young Children Prior to the H1N1 Pandemic. J. Gen. Virol..

[107] Corbett K.S., Edwards D.K., Leist S.R., Abiona O.M., Boyoglu-Barnum S., Gillespie R.A., Himansu S., Schäfer A., Ziwawo C.T., DiPiazza A.T. (2020). SARS-CoV-2 mRNA Vaccine Design Enabled by Prototype Pathogen Preparedness. Nature.

[108] McLellan J.S., Chen M., Joyce M.G., Sastry M., Stewart-Jones G.B.E., Yang Y., Zhang B., Chen L., Srivatsan S., Zheng A. (2013). Structure-Based Design of a Fusion Glycoprotein Vaccine for Respiratory Syncytial Virus. Science.

[109] McLellan J.S., Chen M., Leung S., Graepel K.W., Du X., Yang Y., Zhou T., Baxa U., Yasuda E., Beaumont T. (2013). Structure of RSV Fusion Glycoprotein Trimer Bound to a Prefusion-Specific Neutralizing Antibody. Science.

